# Detection of IncN‐pST15 one‐health plasmid harbouring *bla*_KPC‐2_ in a hypermucoviscous *Klebsiella pneumoniae* CG258 isolated from an infected dog, Brazil

**DOI:** 10.1111/tbed.14006

**Published:** 2021-02-21

**Authors:** Fábio P. Sellera, Bruna Fuga, Herrison Fontana, Fernanda Esposito, Brenda Cardoso, Sibele Konno, Carla Berl, Mariana H. Cappellanes, Marcia Cortez, Marcelo Ikeda, César M. de Souza, Louise Cerdeira, Nilton Lincopan

**Affiliations:** ^1^ Department of Internal Medicine School of Veterinary Medicine and Animal Science University of São Paulo São Paulo Brazil; ^2^ One Health Brazilian Resistance Project (OneBR) São Paulo Brazil; ^3^ Department of Clinical Analysis School of Pharmacy Universidade de São Paulo São Paulo Brazil; ^4^ Department of Microbiology Institute of Biomedical Sciences University of São Paulo São Paulo Brazil; ^5^ PetCare Veterinary Hospital São Paulo Brazil; ^6^ Department of Infectious Diseases Central Clinical School Monash University Clayton Vic. Australia

**Keywords:** carbapenemase, global priority pathogens, one health, pets, plasmidome

## Abstract

The emergence and rapid spread of carbapenemase‐producing Enterobacterales represents a serious public health concern. Critically, these global priority bacteria have begun to be reported in companion animals, implying a potential risk of cross‐transmission between humans and pets. Using long‐read (MinION) and short‐read (Illumina) sequencing technologies, we have identified and characterized a hypermucoviscous KPC‐2‐producing *Klebsiella pneumoniae* strain belonging to the high‐risk international clone ST11/CG258, in a dog with urinary tract infection. Strikingly, the *bla*_KPC‐2_ gene was carried by a 54‐kb IncN plasmid assignated to ST15, which shared 99.8 and 96.8% pairwise identity with IncN‐pST15 plasmids from human and environmental *K. pneumoniae* strains, respectively; all come from an area with high endemicity of KPC‐2. Our findings suggest that IncN‐pST15 plasmids conferring carbapenem resistance can play as important a role as clonal transmission of *K. pneumoniae*, representing another major challenge for One Health.

## 1 INTRODUCTION

Epidemiological studies have revealed that carbapenemase‐producing Enterobacterales have emerged in healthy and sick animals, and community settings (Kelly et al., 2017; Wang et al., 2020; Zhang et al., 2019), implying a potential risk of transmission of these pathogens between humans and companion animals (Grönthal et al., 2018; Sellera & Lincopan, 2019). Additionally, the transfer of carbapenems resistance genes can be facilitated by mobile genetic elements (*e.g*. plasmids and transposons), which is a concerning possibility (Baquero et al., 2019; Brandt et al., 2019).

KPC family has been the most widespread of all carbapenemases associated with Enterobacterales (van Duin & Doi, 2017). The occurrence of KPC‐producing bacteria in human hospital settings has rendered nosocomial infections particularly difficult to treat or even untreatable (Wang et al., 2016). To date, the identification of KPC producers in companion animals has been sporadically reported from dogs in Brazil (KPC‐2‐producing *Escherichia coli*) and United States (KPC‐4‐producing *Enterobacter xiangfangensis*) (Daniels et al., 2018; Sellera et al., 2018).

In this study, under a ‘One Health’ view, we report the identification of a KPC‐2‐positive *Klebsiella pneumoniae* belonging to the international high‐risk clone sequence type 11/clonal group 258 (ST11/CG258) in a dog suffering from urinary tract infection, highlighting that IncN‐pST15 plasmids carrying *bla*_KPC‐2_ genes are spreading among human, animal and environmental clonally unrelated *K. pneumoniae* strains.

## 2 MATERIALS AND METHODS

In 2019, during a Brazilian surveillance study (OneBR project), conducted to characterize the burden of antimicrobial resistance associated with critical WHO priority pathogens, a carbapenem‐resistant *K. pneumoniae* strain (PVT01) identified by BD Phoenix (BD Diagnostics, Sparks, MD, USA) was isolated from a urine culture of a 9‐year‐old female Spitz dog suffering from urinary tract infection.

Antimicrobial susceptibility testing was performed by the disc diffusion and/or Etest methods according to Clinical and Laboratory Standards Institute methods (CLSI, 2018, 2020). The antibiotics tested were amoxicillin/clavulanic acid, aztreonam, cefotaxime, ceftriaxone, cefepime, cefoxitin, ceftiofur, ciprofloxacin, enrofloxacin, nalidixic acid, chloramphenicol, amikacin, gentamicin, ertapenem, imipenem, meropenem, sulfamethoxazole/trimethoprim and tetracycline. Colistin susceptibility testing was performed by broth microdilution method according to European Committee on Antimicrobial Susceptibility Testing (EUCAST, 2021) guidelines. ESBL production was screened by the double‐disc synergy test (DDST) (Drieux et al., 2008), whereas phenotypic detection of KPC enzyme was performed by the combined disc test using imipenem disc supplemented with aminophenylboronic acid (Tsakris et al., 2011). In addition, PVT01 strain was screened for hypermucoviscosity by string test (Shon et al., 2013).

Total genomic DNA was extracted and sequenced using long‐read (MinION, Oxford Nanopore) and short‐read (NextSeq, Illumina) sequencing technologies. Hybrid de novo assembly was performed using Unicycler v0.4.8 (https://github.com/rrwick/Unicycler), whereas Mlplasmids (https://sarredondo.shinyapps.io/mlplasmids/) was used to predict plasmid and chromosome‐derived sequences (Arredondo‐Alonso et al., 2018). Genome sequences were annotated with NCBI PGAP v.3.2 (http://www.ncbi.nlm.nih.gov/genome/annotation_prok/). ABRicate v0.9.8 (https://github.com/tseemann/abricate), with ResFinder 4.1 (https://cge.cbs.dtu.dk/services/ResFinder/) and PlasmidFinder 2.1 (https://cge.cbs.dtu.dk/services/PlasmidFinder/) databases, and Kleborate (https://github.com/katholt/Kleborate) were used for prediction of resistome, plasmidome, species confirmation, multilocus sequence type (ST), virulence loci, and K (capsule) and O antigen (LPS) serotypes (Lam et al., 2018; Wick et al., 2018; Wyres et al., 2016). The nucleotide sequences of *K. pneumoniae* strain PVT01 were deposited at GenBank under accession number JABSUB000000000.1.

## 3 RESULTS AND DISCUSSION

The PVT01 strain exhibited a multidrug‐resistant (MDR) profile (Magiorakos et al., 2012) to amoxicillin/clavulanic acid, aztreonam, ceftriaxone, ceftazidime, cefoxitin, cefotaxime, cefepime, ceftiofur, ertapenem, imipenem, meropenem, amikacin, gentamicin, sulfamethoxazole/trimethoprim, enrofloxacin, ciprofloxacin, nalidixic acid and chloramphenicol, remaining susceptible to tetracycline and colistin (Table 1). ESBL and carbapenemase production were confirmed by the phenotypic tests. Additionally, the PVT01 strain displayed a hypermucoviscous phenotype, as defined by a positive string test (*i.e*. viscous filament ≥ 5 mm in length).

**TABLE 1:** Susceptibility profile and genomic features of KPC‐2‐producing *Klebsiella pneumoniae* strain isolated from an infected dog in Brazil

Susceptibility profile^a^	
Amoxicillin/clavulanic acid	R
Aztreonam	R
Cefotaxime	R
Ceftriaxone	R
Ceftazidime	R
Ceftiofur	R
Cefoxitin	R
Cefepime	R
Ertapenem	R
Imipenem (MIC mg/L)	R (>32)
Meropenem (MIC mg/L)	R (>32)
Amikacin (MIC mg/L)	R (64)
Gentamicin (MIC mg/L)	R (>256)
Sulfamethoxazole/trimethoprim	R
Nalidixic acid	R
Enrofloxacin	R
Ciprofloxacin	R
Chloramphenicol	R
Tetracycline	S
Colistin (MIC mg/L)	S (2)
Molecular epidemiology	
MLST (ST/CG)^b^	11/258
K‐locus	KL15
*wzi*	50
Serotype	O4
Resistome	
β‐lactams	*bla*_KPC−2_, *bla*_CTX‐M−15_, *bla*_LAP−2_, *bla*_OXA−1_, *bla*_SHV−11_
Quinolones	*aac(6')‐Ib‐cr*, *oqxA*, *oqxB*, *qnrS1*, *gyrA* (S83I), *parC* (S80I)
Aminoglycosides	*aac(3)‐IIa*, *aadA2*, *aph(3')‐Ia*
Sulfamethoxazole	*sul1*
Trimethoprim	*dfrA12*
Fosfomycin	*fosA*
Macrolides	*mphA*
Chloramphenicol	*catB4*
Virulome	
Yersiniabactin siderophore	*ybt*, *fyuA*, *irp*
Plasmidome	
Inc‐type [size, kb]^c^	IncFIB(K) [168], IncN [54], Col4401‐like [76]
GenBank accession number	JABSUB000000000.1

^a^ Susceptibility profiles were determined using the CLSI guideline (CLSI, 2020). For ceftiofur, enrofloxacin and colistin, resistance profiles were determined using veterinary CLSI (CLSI, 2018) and EUCAST 2021 (https://www.eucast.org/) guidelines, respectively.

^b^ MLST, Multi‐Locus Sequence Typing; ST, sequence type; CG, clonal group.

^c^ The IncFIB(K) plasmid, named pPVT01_P1, harboured *bla*_CTX‐M‐15_, *aac(3)‐IIa*, *aadA2*, *aph(3')‐Ia*, *mphA*, *sul* and *dfrA12*, whereas Col4401‐like plasmid (pPVT01_P2) harboured *bla*_OXA‐1_, *bla*_LAP‐2_, *qnrS1* and *aac(6')‐Ib‐cr* resistance genes.

Resistome analysis revealed a MDR genotype to β‐lactams, quinolones, aminoglycosides, sulfamethoxazole/trimethoprim, fosfomycin, macrolides and chloramphenicol (Table 1). Moreover, genes encoding for yersiniabactin siderophore synthesis (*ybt*, *fyuA* and *irp* genes) (Paczosa & Mecsas, 2016), and KL15 (*wzi*50) and O4 loci were identified (Wyres et al., 2016).

**FIGURE 1:**
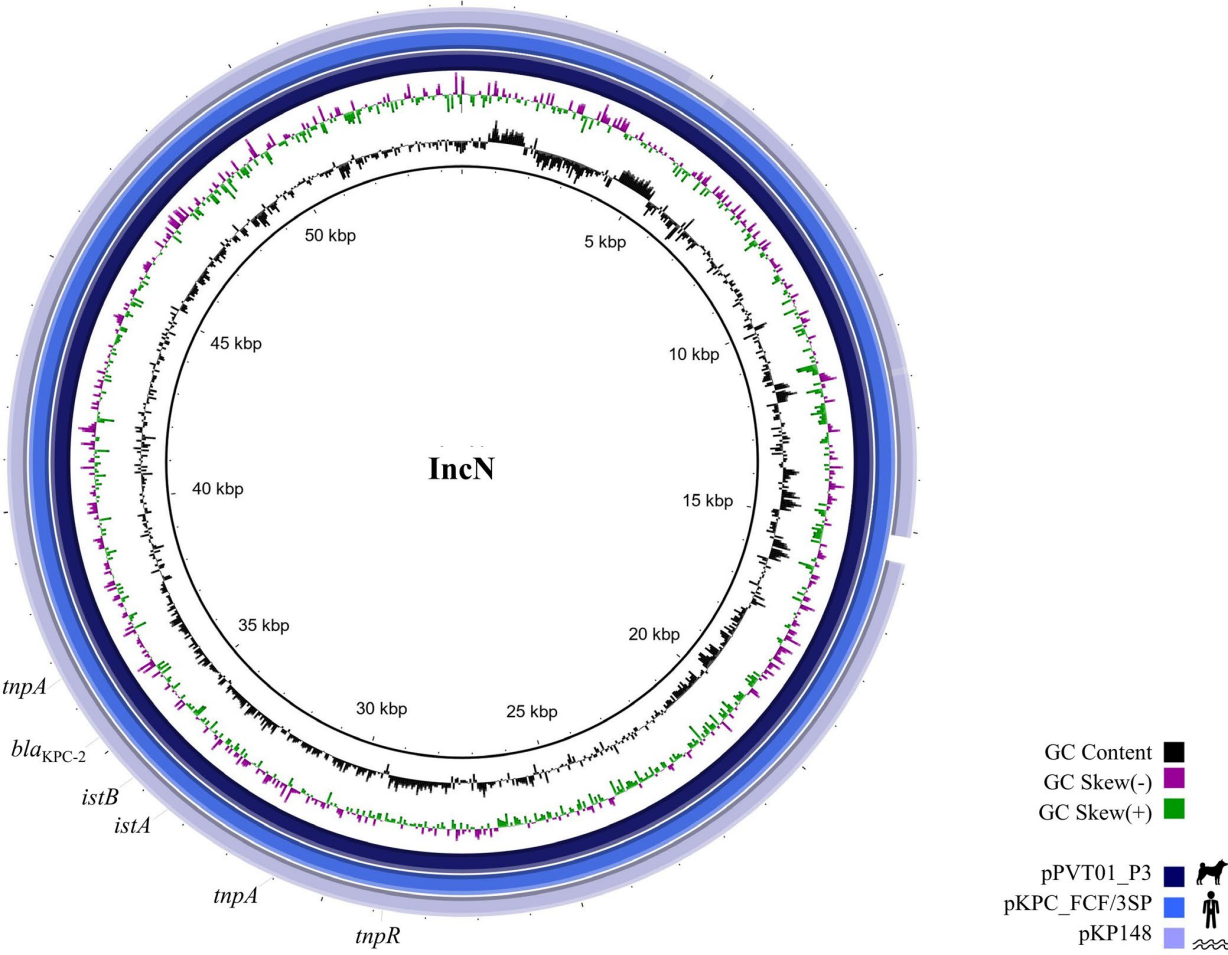
BRIG comparative analysis of pPVT01_P3 IncN‐pST15 plasmid harbouring *bla*_KPC‐2_, from a *Klebsiella pneumoniae* belonging to ST11/CG258 isolated from a dog suffering from urinary tract infection, with two closely related *bla*_KPC‐2_‐positive IncN‐pST15 plasmids from human (pKPC_FCF/3SP, GenBank accession number: CP004367.2) and environmental (pKP148 plasmid, GenBank accession number: KX062091.1) *K. pneumoniae* strains. The coloured rings denote similarity between the plasmid sequences

Hybrid assembly revealed three resistance plasmids: IncFIB(K) (168‐kb), IncN (54‐kb) and Col4401‐like (76‐kb). The IncFIB(K) plasmid, named pPVT01_P1, harboured *bla*_CTX‐M‐15_, *aac(3)‐IIa*, *aadA2*, *aph(3')‐Ia*, *mphA*, *sul1* and *dfrA12*, whereas Col4401(76‐kb)‐like plasmid (pPVT01_P2) harboured *bla*_OXA‐1_, *bla*_LAP‐2_, *qnrS1* and *aac(6')‐Ib‐cr* resistance genes. Specifically, the *bla*_KPC‐2_ gene was carried by the 54‐kb IncN plasmid (named pPVT01_P3) assignated to ST15 by pMLST typing and located on a Tn*4401* transposon > 99% identical to Tn*4401b* isoform (GenBank accession number: EU176012). The plasmid pPVT01_P3 (GenBank accession number: JABSUB010000003.1) shared 99.8 and 96.8% pairwise identity with pKPC_FCF/3SP and pKP148 IncN‐pST15plasmids (GenBank accession numbers: CP004367.2 and KX062091.1), previously identified in human and environmental *K. pneumoniae* strains belonging to ST442 (Pérez‐Chaparro et al., 2014) and ST437, respectively (Oliveira et al., 2014) (Figure 1). Strikingly, all these *K. pneumoniae* strains come from an area with high endemicity of KPC‐2 (Sampaio & Gales, 2016), highlighting the widespread and adaptation of IncN‐pST15 plasmids carrying *bla*_KPC‐2_ at the human–animal–environment interface (Rada et al., 2020), and addressing a One‐Health implication to the problem of rapid dissemination of KPC‐2‐producing *K. pneumoniae*. In fact, *K. pneumoniae* PVT01 belonged to ST11/CG258, recognized as an international high‐risk clone linked to the epidemiological success of pandemic KPC carbapenemases in nosocomial settings (Bialek‐Davenet et al., 2014; Kelly et al., 2017; Rojas et al., 2017; Wyres & Holt, 2018). Worryingly, adaptation of ST11 to veterinary settings has been documented in European and Asian countries (Donati et al., 2014; Hidalgo et al., 2013; Loncaric et al., 2016; Mairi et al., 2020; Ovejero et al., 2017; Pilo et al., 2015; Schmidt et al., 2020; Wang et al., 2020; Wohlwend et al., 2015; Zhang et al., 2019), with KPC‐2‐positive ST11 only being reported in horse (Wang et al., 2020) and swine (Zhang et al., 2019) in China, so far.

In summary, to the best of our knowledge, this is the first report of KPC‐positive *K. pneumoniae* ST11/CG258 isolated from a pet. The emergence of KPC‐2‐producing bacteria in companion animals is an important public health issue that denotes that pets are a neglected reservoir for critical priority pathogens in the community, and susceptible hosts for acquisition of untreatable or difficult‐to‐treat infections (Abraham et al., 2014; Köck et al., 2018; Pomba et al., 2017; Sellera & Lincopan, 2019). In this regard, IncN‐pST15 plasmids conferring carbapenem resistance can play as important a role as clonal transmission of *K. pneumoniae*, representing another major challenge for One Health. Therefore, surveillance studies should investigate similarities of plasmids circulating at the human–environment–animal interface in addition to clonal transmission.

## CONFLICT OF INTEREST

The authors have no conflict of interest to declare.

## ETHICAL APPROVAL

The authors confirm that the ethical policies of the journal, as noted on the journal's author guidelines page, have been adhered to. No ethical approval was required for this specific study.

## Data Availability

All data generated or used during the study appear in the submitted article. The data that support the findings of this study are available from the corresponding author upon reasonable request. The whole genome nucleotide sequence of the *K. pneumoniae* PVT01 strain is available in the GenBank database under accession number JABSUB000000000.1. Genomic data of *K. pneumoniae* strain PVT01 is also available on the OneBR platform (http://onehealthbr.com/) under the number ID ONE247.
